# Imaging features, diagnosis, and clinical management of hepatic involvement in hereditary hemorrhagic telangiectasia: a case series report and literature review

**DOI:** 10.3389/fmed.2026.1869789

**Published:** 2026-07-13

**Authors:** Qiubo Feng, Qinpan Rao, Jiaqi Duan, Shufeng Fan, Linying Ma

**Affiliations:** Department of Radiology, The Second Affiliated Hospital of Zhejiang Chinese Medical University, Hangzhou, Zhejiang, China

**Keywords:** computed tomography, hepatic arteriovenous malformations, hepatic artery embolization, hereditary hemorrhagic telangiectasia, magnetic resonance imaging, portal vein thrombosis

## Abstract

To explore the clinical characteristics, imaging features, key diagnostic and therapeutic points, and risks of interventional therapy in hereditary hemorrhagic telangiectasia (HHT) with hepatic involvement, so as to improve the ability of clinicians and radiologists in the identification, diagnosis and treatment of this disease. A retrospective analysis was performed on the clinical data of 3 patients with HHT who were admitted to our hospital in the past 5 years and presented with predominant hepatic involvement. Their clinical symptoms, laboratory test results, imaging findings, and treatment outcomes were summarized, and a literature review was conducted for comprehensive analysis. All 3 patients met the Curacao diagnostic criteria for HHT and were found to have diffuse hepatic arteriovenous malformations, mainly presenting as mixed shunts. Computed tomography (CT) and magnetic resonance imaging (MRI) revealed dilated hepatic arteries, early visualization of the portal vein and hepatic vein, diffuse intrahepatic telangiectasias, and abnormal perfusion. One patient was complicated with biloma, and another was associated with portal hypertension and esophagogastric varices. Patient 3 underwent transcatheter hepatic arterial embolization (TAE) due to abdominal pain, and extensive thrombosis of the main portal vein and superior mesenteric vein occurred after the operation. Hepatic involvement in HHT is characterized by diffuse hepatic vascular malformations, with insidious clinical manifestations that are prone to misdiagnosis. Doppler ultrasound is the preferred screening method, while CT/MRI can comprehensively evaluate vascular malformations and related complications. Hepatic arterial embolization has a high risk of inducing portal vein thrombosis, and its routine application should be avoided in strict accordance with international guidelines. Clinicians need to strengthen medical history collection and multimodal imaging evaluation to reduce misdiagnosis and mistreatment.

## Introduction

HHT also known as Rendu-Osler-Weber syndrome, is an autosomal dominant hereditary vascular dysplasia with a global incidence of approximately 1 in 5,000 to 1 in 8,000 (1). Its core pathological changes are telangiectasias and arteriovenous malformations in the skin, mucous membranes and multiple organs (1–3). This disorder is predominantly attributed to mutations in ENG or ACVRL1, whereas hepatic manifestations are characteristically more frequent in HHT type 2 (associated with ACVRL1 mutations) (4). The incidence rates for both men and women are the same, but relevant literature indicates that the frequency of liver involvement in women is 4.5 times that of men (5). Penetrance is age-dependent, increasing with age and reaching up to 97% by the age of 40 (6, 7).

The diagnosis of HHT relies on the Curaçao criteria described by Shovlin et al. (8, 9) (1) spontaneous, recurrent epistaxis; (2) mucocutaneous telangiectasias at characteristic sites (e.g., lips, oral cavity, fingers); (3) visceral involvement (e.g., pulmonary, hepatic, or cerebral arteriovenous malformations (AVMs), gastrointestinal bleeding); and (4) a first-degree relative with HHT. A definite diagnosis requires meeting three or more criteria, while two criteria suggest a possible or suspected diagnosis, and fewer than two make the diagnosis unlikely. Due to the lack of specific early manifestations, the disease is frequently misdiagnosed as liver cirrhosis or common hepatic arteriovenous fistulas (8, 10). HHT-related hepatic vascular malformations are typically characterized by diffuse involvement involving hepatic artery-portal vein, hepatic artery-hepatic vein, and portal vein-hepatic vein shunts. Chronic shunting predisposes patients to serious complications such as high-output heart failure (HOCF), portal hypertension, biliary ischemia, and bilomas (6, 7). Imaging represents the core modality for the diagnosis, characterization, classification, and assessment of complications of this disease. Doppler ultrasonography, CT, MRI, and digital subtraction angiography (DSA) each have distinct advantages; however, knowledge of their characteristic imaging features in clinical practice remains insufficient (11). This has led to inappropriate interventional procedures performed due to poor recognition of these findings, resulting in severe complications. This study retrospectively analyzed the multimodal imaging data of 3 patients with hepatic involvement in HHT, systematically summarized the imaging signs, diagnostic points, and differential diagnosis, and combined with literature review, aiming to provide a reference for accurate imaging diagnosis.

## Case report

Case selection criteria: The study period was from January 2021 to December 2025. Patients treated at our hospital with hepatic arteriovenous malformations confirmed by contrast-enhanced CT/MRI, meeting the Curacao diagnostic criteria, and with complete clinical and imaging data were included. Patients with concomitant primary liver disease, malignant tumors, or incomplete data were excluded.

Imaging methods: Upper abdominal non-enhanced and contrast-enhanced CT was performed using a GE Lightspeed 64-slice helical CT scanner. MRI was performed using a Siemens Avanto 1.5 T MR scanner with multiphase dynamic contrast enhancement. Abdominal ultrasonography was performed using a Toshiba Aplio 500 color Doppler ultrasound system to evaluate the liver and involved arteriovenous systems, including two-dimensional, color, and spectral Doppler modes. Data on baseline characteristics, symptoms, laboratory examinations, multimodal imaging (ultrasound, CT/MRI, DSA), treatment procedures, and follow-up outcomes were collected. The clinical manifestations and outcomes of the three patients are summarized in Table 1.

**Table 1:** Clinical manifestations and outcomes of three HHT patients with hepatic involvement.

Items	Case 1	Case 2	Case 3
Sex/age	Female/65 years	Female/60 years	Female/39 years
Initial symptom	Chronic heart failure	Appendicitis-like abdominal pain	Abdominal distension and epigastric pain
Epistaxis	Present	Present for more than 10 years	Present
Cutaneous telangiectasia	Present	Absent	Slight
Family history	Negative	Positive	Negative
Curacao criteria	3/4	3/4	3/4
Hepatic artery diameter	8.7 mm	8.0 mm	7.0 mm
Shunt type	Mixed type	Mixed type	Mixed type
Baseline complications	Portal hypertension, splenomegaly	Bilioma	Portal hypertension, esophagogastric varices
Treatment regimen	Symptomatic conservative treatment	Symptomatic treatment	Hepatic artery embolization
Postoperative complications	None	None	Extensive thrombosis of the portal vein and superior mesenteric vein
Follow-up duration	54 months	42 months	3 days
Follow-up method	Telephone follow-up	Telephone follow-up	Lost to follow-up after referral
Clinical outcome	Stable	Stable	Portal vein thrombosis, transferred for further treatment
Survival status	Alive	Alive	Lost to follow-up

### Case 1

A 65-year-old woman presented to our hospital in August 2022 with chronic heart failure. The key imaging findings from contrast-enhanced upper abdominal CT and multiphase dynamic contrast-enhanced MRI comprised hepatic arterial dilatation (approximately 8.7 mm in diameter), early opacification of the portal and hepatic veins during the arterial phase, diffuse abnormal hepatic perfusion, portal hypertension, and splenomegaly (Figures 1A–H). Physical examination revealed scattered cutaneous telangiectasias, and the patient reported a history of recurrent epistaxis, no family history. The diagnosis of HHT was confirmed based on clinical criteria. The patient is currently receiving symptomatic treatment for heart failure and undergoing regular follow-up examinations at a local hospital.

**Figure 1:**
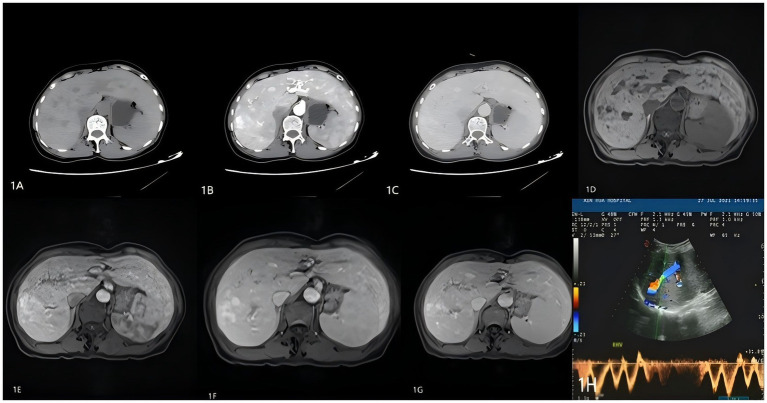
A 65-year-old female presented with chronic heart failure. Contrast-enhanced upper abdominal MRI and CT **(A–G)** showed multiple intrahepatic arteriovenous fistulas, a dilated hepatic artery (approximately 8.7 mm in diameter), tortuous and dilated portal and hepatic veins, and multiple intrahepatic perfusion abnormalities. Doppler ultrasound **(H)** revealed altered intrahepatic blood flow. The patient had a history of recurrent epistaxis, and physical examination showed scattered cutaneous petechiae. The patient is under ongoing follow-up.

### Case 2

A 60-year-old woman presented to our hospital in July 2021 with appendicitis-like abdominal pain. Non-enhanced scan revealed an abnormal density lesion in the right lobe of the liver. Contrast-enhanced multiphase dynamic MRI of the liver shows multiple arteriovenous malformations, diffuse telangiectasias in the arterial phase, and a local non-enhancing hypointense area, suggesting a biloma. Doppler ultrasound indicated abnormal intrahepatic blood vessels and dilated hepatic veins (Figures 2A–C). Upon further inquiry about the medical history, the patient had a history of recurrent epistaxis for more than 10 years and a positive family history. The patient met the confirmed diagnostic criteria for HHT. The patient received symptomatic treatment, and her clinical condition remains stable with regular ongoing follow-up at a local hospital.

**Figure 2:**
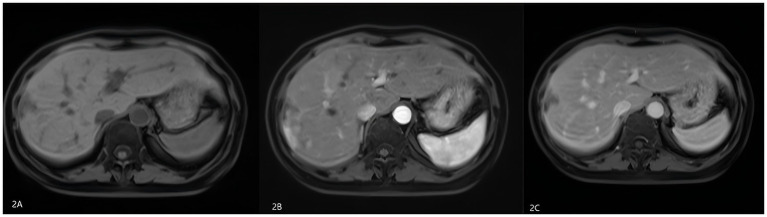
A 60-year-old female presented with appendicitis-like abdominal pain. Contrast-enhanced MRI **(A–C)** demonstrated multiple intrahepatic arteriovenous fistulas, suspicious for associated focal necrotic biloma. The patient had a more than 10-year history of recurrent epistaxis, with a positive family history of similar recurrent epistaxis and multiple hepatic arteriovenous fistulas. The patient is currently in good condition.

### Case 3

A 39-year-old female presented with a 10-day history of abdominal distension and epigastric pain. Key imaging findings on contrast-enhanced CT venography (CTV) and contrast-enhanced multiphase dynamic MRI included early opacification of the portal and hepatic veins during the arterial phase, dilation of the common hepatic artery (approximately 7 mm), and diffuse punctate/nodular enhancement within the liver. The portal venous phase revealed portal vein dilation (1.3 cm), esophagogastric varices, splenomegaly, bowel wall edema, and a small amount of ascites, suggestive of mixed-type hepatic arteriovenous fistulas combined with portal hypertension. Doppler ultrasound demonstrated a mosaic color flow pattern in the hepatic artery. Diagnosis and treatment course: The patient reported partial relief of abdominal pain after administration of antispasmodic drugs. Abdominal pain was therefore suspected to arise from portal hypertension and hepatic arteriovenous fistula, leading to mesenteric ischemia. No family history or other relevant medical history was obtained. Treatment was focused on managing portal hypertension and hepatic arteriovenous fistula. DSA confirmed the presence of both hepatic artery-to-portal vein and hepatic artery-to-hepatic vein shunts. The patient underwent hepatic artery embolization using polyvinyl alcohol (PVA), which initially alleviated the abdominal pain. However, pain recurred 3 days later. Follow-up CTV revealed extensive thrombosis involving the main portal vein, its right and left branches, and the superior mesenteric vein (Figures 3A–H). Upon review of the patient’s history, which included epistaxis and scattered petechiae, but no family history. The diagnosis was revised to HHT. The patient was subsequently transferred to another facility for further management.

**Figure 3:**
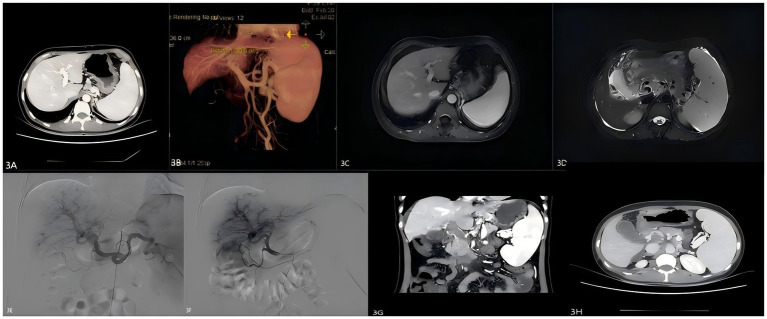
A 39-year-old female presented with abdominal pain. **(A–D)** CT, MR, and CTA demonstrated multiple small patchy areas of abnormal enhancement in the hepatic parenchyma, with early opacification of the portal vein and hepatic vein, marked edema of the gastric wall and ascending colon wall, dilatation of the portal vein and its branches, as well as esophagogastric varices. **(E,F)** DSA revealed diffuse patchy and reticular abnormal enhancement at the hepatic arteriovenous junction, accompanied by early opacification of the hepatic vein and portal vein. **(G,H)** Hepatic portal venous CTV showed extensive thrombosis in the main portal vein, left and right portal branches, and superior mesenteric vein.

## Discussion

Hepatic involvement in HHT is mostly asymptomatic. The prevalence of hepatic involvement in unselected HHT patients evaluated by spiral CT reaches 70%, whereas only 5–8% of patients present with clinical manifestations. The mean age at diagnosis was 48 years (5, 12). Symptom severity in affected patients correlates closely with the anatomic extent of vascular lesions and the presence of arteriovenous shunting. Common clinical manifestations and complications include: HOCF, the most common complication. Blood from the hepatic artery is shunted directly into the hepatic vein, bypassing the hepatic sinusoids, leading to a marked increase in cardiac preload (11). The condition becomes clinically significant when the intrahepatic shunt volume exceeds 20% of cardiac output. The cardiac index is usually elevated to 4.2–7.3 L/min・m^2^ (normal range 2.5–4.0), manifestations include dyspnoea, a hepatic bruit and lower extremity edema (13). Garcia-Tsao followed eight symptomatic patients with HOCF; after a median follow-up of 24 months, three improved, four remained stable on medication, and one died (post-embolization bile duct necrosis and sepsis) (11). Portal hypertension or nodular regenerative hyperplasia, arterial blood flows directly into the portal venous system, increasing portal venous flow; regenerating nodules compress small portal vein branches, causing increased pre-sinusoidal resistance. Clinical manifestations include ascites, esophageal and gastric varices, and imaging findings resembling those of pseudo-cirrhosis, but liver function is usually normal (12, 14). Biliary ischemic necrosis, the most severe complication. High-flow arteriovenous shunting “steals” blood from the peribiliary plexus, leading to biliary ischemia. Severe cases can result in hepatocyte necrosis, hepatic hemorrhage and bile leakage (15). Garcia-Tsao followed five patients with biliary complications; after a median follow-up of 30 months, two improved, one remained unchanged, one developed secondary heart failure, and one died after failed liver transplantation (11, 14). In our group of three patients, the presenting symptoms were abdominal pain, appendicitis-like symptoms and chronic heart failure, respectively. Two of them did not have typical initial manifestations of hepatic HHT, suggesting that this condition is highly susceptible to misdiagnosis. Imaging is an important method for the diagnosis of HHT.

### Multimodal imaging evaluation

#### Doppler ultrasonographic findings

Due to its safety, tolerability, and cost-effectiveness, Doppler ultrasound is the preferred screening modality for hepatic arteriovenous malformations (11). Caselitz et al. (16) proposed two major criteria: dilation of the common hepatic artery (>7 mm) and intrahepatic arterial hypervascularity. Minor criteria include a hepatic artery peak systolic velocity (PSV) > 110 cm/s, a resistive index (RI) of the proper hepatic artery <0.60, a portal vein PSV > 25 cm/s, and tortuosity of the extrahepatic hepatic artery. A diagnosis of hepatic vascular malformations in HHT is established when two major criteria are met, or when one major criterion is accompanied by at least two minor criteria. Dilated intrahepatic arterial branches appear as tubular structures running parallel to the portal vein branches, creating the “parallel channel sign.” In severe cases, these vessels exhibit a tortuous and tangled appearance. Pulsed and color Doppler ultrasound allow for the rapid analysis of hemodynamic patterns in hepatic vascular malformations. This includes qualitative parameters such as flow direction and turbulence; quantitative parameters like angle-corrected flow velocities (peak hepatic artery velocity, mean portal vein velocity, and peak diastolic hepatic vein velocity); and semi-quantitative parameters including the RI and pulsatility index (PI) (17). However, ultrasonography is highly operator-dependent. Consequently, CT and MRI are recommended as essential investigations, particularly when coexisting hepatic lesions or severe arteriovenous malformations are suspected.

#### Imaging characteristics on CT and MRI

Contrast-enhanced multi-detector CT, contrast-enhanced MRI and reconstructed images (volume rendering (VR), maximum intensity Projection (MIP)) can clearly demonstrate the complex hepatic vascular anomalies, shunt types and abnormal liver parenchymal perfusion typical of HHT, while also assessing the spleen, oesophagogastric varices and other collateral circulations (14, 18–20). (1) Abnormal intrahepatic vascular shunting: a typical manifestation of hepatic involvement in HHT. It is classified into three types: hepatic artery-portal vein shunt (the most common type), hepatic artery-hepatic vein shunt, and portal vein-hepatic vein shunt. (2) Telangiectasia: Characteristically diffuse, these appear as scattered, patchy high-density areas in the arterial phase, which fade to near-isodensity relative to the surrounding parenchyma by the equilibrium phase. The literature reports that the incidence rate is 90%. In this study, all three patients exhibited this symptom. (3) Hepatic arterial dilatation and tortuosity: The arterial phase reveals dilated and tortuous hepatic arteries in the hepatic hilum and intrahepatic regions. MIP reconstructions optimally demonstrate the dilated, tortuous, and disorganized hepatic arterial vasculature, with MIP being particularly effective for visualizing intrahepatic vascular malformation clusters. VR images provide three-dimensional visualization of the dilated and tortuous hepatic arteries. (4) Focal hepatic lesions: HHT can induce diffuse or partial hepatocellular regeneration, manifesting as nodular regenerative hyperplasia (NRH) or focal nodular hyperplasia (FNH). The prevalence of FNH is approximately 100-fold higher in HHT patients compared to the general population. The hallmark of FNH is delayed enhancement of a central fibrous scar. Despite the presence of multiple hyperplastic nodules, liver function often remains preserved, creating a diagnostic challenge that may mimic pseudocirrhosis. (5) Biliary abnormalities: These manifest as biliary dilation and strictures, or localized biliary ectasia. Ischemia resulting from abnormal hepatic vascular shunts, or compression by tortuous and dilated hepatic arteries, may lead to necrotizing cholangitis with biloma formation. This represents the most severe complication of hepatic vascular malformations in HHT. Imaging typically reveals solitary or multiple ill-defined hypodense lesions with no enhancement. One patient in our study presented with this complication.

As the diagnostic “gold standard” for hepatic vascular malformations, DSA-specifically early arterial phase celiac angiography—depicts characteristic findings such as tortuous, dilated hepatic arteries and diffuse telangiectasias (21). It also visualizes arteriovenous shunting, evidenced by the premature filling of hepatic or portal veins, and allows for the detection of concurrent vascular anomalies.

Doppler ultrasound is safe, non-invasive, and repeatable, serving as the first-line screening tool (14). It can rapidly identify hepatic arterial dilatation, high-resistance low-velocity blood flow, and intrahepatic hyperperfusion. Contrast-enhanced CT and CT angiography (CTA) can directly and sensitively demonstrate intrahepatic abnormal lesions. MIP and VR images provide more intuitive and clear visualization of vascular abnormalities, and are commonly used in clinical diagnosis, evaluation, screening, and follow-up of HHT. Because CT carries risks of X-ray radiation and adverse reactions to contrast agents, contrast-enhanced multiphase dynamic MRI can be used as the preferred modality when follow-up examination by an ultrasound expert is not available. Multi-sequence MRI can clearly depict hepatic vascular malformations as well as biliary abnormalities, which is valuable for the early diagnosis of HHT without concerns about ionizing radiation. However, it has limitations such as relatively long examination duration, potential respiratory artifacts, and specific contraindications. DSA is the gold standard for the diagnosis of arteriovenous fistulas. Nevertheless, it is an invasive and technically demanding procedure, making it unsuitable for routine follow-up and large-scale screening.

Depending on the patient’s specific circumstances, the follow-up monitoring plan is divided into the following six scenarios (14, 22–25): (1) Asymptomatic patients without hepatic vascular malformations. Follow-up every 5–10 years, or every 3–5 years based on clinical risk stratification, is recommended. Doppler ultrasound is the preferred examination. (2) Patients with hepatic vascular malformations who are asymptomatic. Follow-up every 1 to 2 years is recommended. The preferred examinations are Doppler ultrasound and echocardiography. (3) All patients diagnosed with hepatic vascular malformations, regardless of symptom status. Echocardiography should be performed at the time of diagnosis to determine the cardiac index and pulmonary artery pressure, thereby establishing baseline data. (4) Patients at intermediate-to-high risk. Doppler ultrasound every 2 years is recommended. (5) High-risk patients. Doppler ultrasound every 6 months and echocardiography annually are recommended. (6) Patients who are symptomatic. The subsequent treatment plan should be individualized based on the specific type of complication and evaluated and managed by a multidisciplinary team.

### Differential diagnosis

The differential diagnosis includes: (1) Cirrhosis, which is characterized by the absence of diffuse arteriovenous shunting, significant hepatic arterial dilation, or early venous filling (26); (2) Idiopathic hepatic arteriovenous fistula, which tends to be focal and lacks the systemic telangiectasias and family history associated with HHT (10), (3) Hypervascular hepatic tumors, which present with a distinct mass effect and enhancement pattern but lack diffuse shunting (27) (Table 2).

**Table 2:** Differential diagnosis of hepatic lesions.

Feature	Hepatic involvement in HHT	Liver cirrhosis	Idiopathic hepatic arteriovenous fistula
Core pathology	Diffuse arteriovenous malformation (AVM)	Hepatocyte necrosis and pseudolobule formation	Focal vascular communication
Hepatic artery	Thickened (diameter > 7 mm), tortuous	Generally not thickened	Local thickening, no diffuse changes
Venous opacification	Early visualization of the portal vein/hepatic vein in the arterial phase	Portal hypertension, collateral circulation	Early opacification limited to the fistula site
Associated symptoms	Epistaxis, telangiectasia, family history	Palmar erythema, spider angiomas, ascites	Typically no systemic symptoms
Treatment contraindication	Blind hepatic artery embolization is strictly prohibited	Case-dependent	Embolization is feasible

### Treatment principles and complication alerts

According to the Second International Guidelines for the Diagnosis and Treatment of Hereditary Hemorrhagic Telangiectasia (24), hepatic artery embolization should be avoided in any patient with confirmed or suspected HHT and hepatic arteriovenous fistulas, as it is only a temporary procedure associated with a high recurrence rate. Intensive treatment should be administered to patients with complex and/or symptomatic disease and tailored according to the type of complications. For symptomatic complications of hepatic HHT, especially those with refractory high-output heart failure, biliary ischemia, or complex portal hypertension, referral for liver transplantation should be considered (28). However, liver transplantation is limited by high surgical difficulty, frequent postoperative complications, and the shortage of available donor livers (29). A systematic review by Riera-Mestre et al. included 83 worldwide patients who underwent liver transplantation for hepatic HHT, with a 6-year survival rate of 88% (5). Liver transplantation is technically demanding, associated with numerous postoperative complications, and donor livers are difficult to obtain; therefore, its implementation remains limited.

Although hepatic artery embolization is associated with high rates of complications and mortality, it may still be an option for selected patients with complications such as high-output cardiac failure and portal hypertension. The procedure is performed in multiple stages (one to five sessions), the most used one consists of an initial embolization of vascular bed with a mixture of polyvinyl alcohol followed by embolization with microcoils. Arterial branches of right and left lobe have to be embolized in different sessions (14). Ajay Chavan et al. followed 20 patients with HHT who underwent hepatic artery embolization for 17 years and found an overall long-term mortality rate of 30% among the 20 patients (29). This is comparable to the 20% mortality rate reported by Lerut and colleagues in 40 patients who underwent liver transplantation (30). The most common post-procedural complications are biliary or hepatic necrosis, occurring in approximately 20–60% of cases (29). In our case, extensive portal vein thrombosis developed. The main contributing factors were analyzed as follows (31–33): we performed direct embolization of the right hepatic artery without stepwise embolization; after embolization, hepatic arterial perfusion declined sharply, leading to portal venous compensatory flow stasis; sinusoidal endothelial injury activated the coagulation system; and the underlying vascular structural abnormalities inherent to HHT collectively promoted thrombus formation. Although hepatic artery embolization can be used to treat hepatic vascular malformations, it requires multi-stage, stepwise embolization. Given that some patients may develop fatal complications, hepatic artery embolization is more suitable for symptomatic patients with hepatic arteriovenous fistulas who are not candidates for liver transplantation. However, embolization is strictly contraindicated in patients complicated by cholangitis or biloma to avoid precipitating biliary ischemia (30).

For some patients with HHT complicated by high-output heart failure and portal hypertension, hepatic artery ligation may also be a potential treatment. The procedure involves reducing the total hepatic artery diameter by one-third to one-half, which may include the lobar arteries. Preoperative contrast-enhanced CT angiography should be routinely performed to understand the intra and extrahepatic vascular anatomy, and intraoperative Doppler ultrasound can be used as an adjunct (34). Liu et al. (35) followed 35 patients with HHT who underwent hepatic artery ligation for 6 to 113 months. Only two patients developed cholangitis, one patient died within a median follow-up of 50 months, and the remaining patients showed symptom improvement and improvement in all aspects of quality of life. At Qilu Hospital of Shandong University, two patients with HHT who underwent laparoscopic hepatic artery ligation were followed up for several years, and a marked improvement in their quality of life was observed (34). Therefore, based on previous experience, angiography can be used to plan transarterial embolization for hepatic vascular malformations. Peripheral staged embolization of hepatic vascular malformations is an effective transarterial treatment strategy. However, because some patients may develop fatal complications, this approach is more suitable for severely symptomatic patients with hepatic arteriovenous fistulas who are not candidates for or unable to undergo liver transplantation.

Among patients followed at HHT centers after systematic screening and treatment, life expectancy did not differ significantly from that in the general population (75.9 years in the HHT cohort versus 79.3 years in the control group) (36). However, survival was significantly lower in patients with AVMs than in those without visceral AVMs (*p* = 0.017) (36). Moreover, patients with isolated hepatic vascular malformations (HVM) had significantly poorer survival than those without HVM (36). In addition, studies have demonstrated that HHT-related liver failure is associated with a higher risk of depression (3.1-fold), anxiety (3.0-fold), and fatigue (9.6-fold) (37). Therefore, enhanced disease awareness, early screening and diagnosis, and more effective interventions are critical to improving outcomes in patients with HHT.

This study has several limitations: it is a single-center, small-sample study, and none of the patients underwent genetic testing. It is difficult to draw any general conclusions regarding clinical characteristics and treatment outcomes. Studies in larger patient populations are still needed to further confirm the efficacy.

### Causes of misdiagnosis and key preventive points

(1) A superficial diagnosis of “hepatic arteriovenous fistula” alone is insufficient. For any patient with hepatic lesions, a detailed medical history, including family history and personal history, should be carefully obtained. (2) Imaging that focuses only on focal lesions without recognizing features of diffuse vascular malformations is inadequate. Radiologists should broaden their diagnostic approach; for patients with unexplained vascular malformations, clinical history should be promptly inquired to expand the differential diagnosis. (3) Treatment decisions should involve multidisciplinary collaboration and a multidisciplinary assessment. Before any intervention for a hepatic arteriovenous fistula, the relevant specialty guidelines should be carefully reviewed, and routine interventional protocols should not be applied blindly.

## Conclusion

In summary, HHT is frequently misdiagnosed or diagnosed with significant delay, risking life-threatening complications, unnecessary invasive procedures, and increased healthcare burdens. While Doppler ultrasound is the preferred screening tool, CT and MRI are vital for characterizing the full spectrum of vascular anomalies. Clinicians must maintain a high index of suspicion for HHT in cases of suspected hepatic arteriovenous fistula, regardless of atypical presentation. A rigorous approach involving detailed history taking, multimodal imaging, and multidisciplinary collaboration is imperative to ensure accurate diagnosis and optimize patient outcomes.

## Data Availability

The original contributions presented in the study are included in the article/Supplementary material, further inquiries can be directed to the corresponding authors.
