# Supplementary material for: Imaging features, diagnosis, and clinical management of hepatic involvement in hereditary hemorrhagic telangiectasia: a case series report and literature review
Source: Front Med (Lausanne). 2026 Jul 13;13:1869789. doi: 10.3389/fmed.2026.1869789 (PMC13404187; doi:10.3389/fmed.2026.1869789)
Supplement: Supplementary file 1 [file Data_sheet_1.pdf]

CARE Checklist of information to include when writing a case report

CARE case report guidelines

(cc) BY-NC-ND

Topic	Item	Checklist item description	Reported on Line
Title	1	The diagnosis or intervention of primary focus followed by the words "case report"	we have achieved
Key Words	2	2 to 5 key words that identify diagnoses or interventions in this case report, including "case report"	we have achieved
Abstract (no references)	3a	Introduction: What is unique about this case and what does it add to the scientific literature?	we have contained
	3b	Main symptoms and/or important clinical findings	we have achieved
	3c	The main diagnoses, therapeutic interventions, and outcomes	we have achieved
	3d	Conclusion—What is the main "take-away" lesson(s) from this case?	we have contained
Introduction	4	One or two paragraphs summarizing why this case is unique (**may include references**)	we have achieved
Patient Information	5a	De-identified patient specific information.	we have achieved
	5b	Primary concerns and symptoms of the patient.	we have contained
	5c	Medical, family, and psycho-social history including relevant genetic information	we have contained
	5d	Relevant past interventions with outcomes	we have discussed
Clinical Findings	6	Describe significant physical examination (PE) and important clinical findings.	we have achieved
Timeline	7	Historical and current information from this episode of care organized as a timeline	we have achieved
Diagnostic Assessment	8a	Diagnostic testing (such as PE, laboratory testing, imaging, surveys).	we have contained
	8b	Diagnostic challenges (such as access to testing, financial, or cultural)	we have discussed
	8c	Diagnosis (including other diagnoses considered)	we have contained
	8d	Prognosis (such as staging in oncology) where applicable	we have achieved
Therapeutic Intervention	9a	Types of therapeutic intervention (such as pharmacologic, surgical, preventive, self-care)	we have contained
	9b	Administration of therapeutic intervention (such as dosage, strength, duration)	we have contained
	9c	Changes in therapeutic intervention (with rationale)	we have contained
Follow-up and Outcomes	10a	Clinician and patient-assessed outcomes (if available)	we haven't
	10b	Important follow-up diagnostic and other test results	we have contained
	10c	Intervention adherence and tolerability (How was this assessed?)	we have contained
	10d	Adverse and unanticipated events	we have contained
Discussion	11a	A scientific discussion of the strengths AND limitations associated with this case report	we have contained
	11b	Discussion of the relevant medical literature **with references**.	we have achieved
	11c	The scientific rationale for any conclusions (including assessment of possible causes)	we have contained
	11d	The primary "take-away" lessons of this case report (without references) in a one paragraph conclusion	we have contained
Patient Perspective	12	The patient should share their perspective in one to two paragraphs on the treatment(s) they received	we haven't
Informed Consent	13	Did the patient give informed consent? Please provide if requested	Yes ☑ No ☐
